# Effect of Infant Formula Made With Milk Free of A1‐Type β‐Casein on Growth and Comfort: A Randomized Controlled Trial

**DOI:** 10.1002/fsn3.70606

**Published:** 2025-07-15

**Authors:** Jing Li, Tingting Yang, Xiaoyang Sheng

**Affiliations:** ^1^ Department of Neonatology, School of Medicine, Shanghai First Maternity and Infant Hospital Tongji University Shanghai China; ^2^ Shanghai Weierkang Pediatric Outpatient Department Shanghai China; ^3^ Department of Developmental Behavioral Pediatric & Children Healthcare, School of Medicine, Xinhua Hospital Shanghai Jiao Tong University Shanghai China

**Keywords:** beta‐casein, crying, dermatitis, growth, immune tolerance, infant formula, quality of life

## Abstract

Consumption of milk free of A1‐type β‐casein (A1PF) can result in reduced gastrointestinal/inflammatory symptoms versus conventional milk (containing A1‐ and A2‐type β‐casein; CON); however, the comparative effects of infant formula made with these milks are unknown. This study compared growth, gastrointestinal tolerance, and crying frequency/duration in healthy Chinese infants aged 3–4 months who consumed both breastmilk and A1PF or CON infant formula. This open‐label, randomized, controlled trial conducted between July and November 2023 evaluated anthropometric measurements and comfort (Infant Gastrointestinal Symptom Questionnaire [IGSQ] and frequency of crying) of mixed‐fed infants who also consumed A1PF or CON infant formula. Eczema/dermatitis severity, quality of life, satisfaction with the formula, and adverse events were also assessed. In the A1PF (*n* = 140) and CON groups (*n* = 140), the mean (standard deviation) ages were 100.83 (15.01) and 99.28 (13.15) days, respectively. There were no significant between‐group differences in any anthropometric measurements. At Weeks 2 and 4, the A1PF group had significantly lower mean daily total IGSQ scores; lower mean stooling, spitting up/vomiting, crying, and fussiness domain scores; and fewer daily crying episodes than the CON group (*p* < 0.05). From satisfaction surveys, a significantly higher percentage of the A1PF group had relieved gastrointestinal symptoms, reduced vomiting after feeding, improved digestion, and better stool characteristics (*p* < 0.05). Both formulas were generally well tolerated. Among mixed‐fed infants, those who consumed A1PF infant formula had the same growth as those fed CON infant formula, and improved comfort (including relieved gastrointestinal symptoms and fewer crying episodes).

**Trial Registration:** ClinicalTrials.gov, NCT06256094

## Graphical abstract

We compared growth, gastrointestinal tolerance, and crying frequency/duration in healthy Chinese infants aged 3–4 months who consumed both breastmilk and formula free of A1‐type β‐casein (A1PF) or conventional infant formula (CON). At Weeks 2 and 4, the A1PF group had significantly lower mean daily total Infant Gastrointestinal Symptom Questionnaire (IGSQ) scores; lower mean stooling, spitting up/vomiting, crying, and fussiness domain scores; and lower mean number of daily crying periods than the CON group (*p* < 0.05). Both formulas were generally well tolerated, but infants who consumed A1PF infant formula had improved comfort as measured by relieved gastrointestinal symptoms and fewer crying periods.

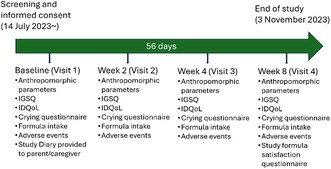

## 1 Introduction

Nutrition during infancy can influence later life outcomes (Ames et al. 2023). As parents/caregivers may include formula as part of infant nutrition, it is imperative to continually improve the quality of these products to ensure healthy early growth and normal development (Bakshi et al. 2023; Ren et al. 2022).

Recent developments to infant formula include supplementation with prebiotics, probiotics, micronutrients, and other additions to support infant gastrointestinal comfort (Bakshi et al. 2023; Haiden et al. 2024). However, it is also important to consider the base ingredient of infant formula, which is primarily cows' milk (Bakshi et al. 2023). Cows' milk comprises approximately 3.4% (weight/volume) protein, of which 30% is β‐casein (Kamiński et al. 2007; Noni 2008). There are at least a dozen β‐casein variants, which evolved from the original A2 β‐casein variant (Cieślińska et al. 2022). The A1‐type variants of β‐casein have a histidine instead of a proline residue at position 67, leading to an increased propensity to yield β‐casomorphin‐7 (BCM‐7) during digestion (Noni 2008; Duarte‐Vázquez et al. 2017; Pal et al. 2015). BCM‐7 is a bioactive peptide that binds to μ‐opioid receptors and limits the production of the antioxidant glutathione (Trivedi et al. 2014; Deth et al. 2016), leading to inflammation. This can result in a range of gastrointestinal and inflammatory effects on the body (Pasi et al. 1993; Ul Haq et al. 2015; Jianqin et al. 2016; He et al. 2017; Woodford 2021) including in infants (Sadler and Smith 2013). Apnoea in sudden infant death syndrome has been linked to BCM‐7 (Sun et al. 2003; Hedner and Hedner 1987), with higher serum levels of BCM‐7 and its derivatives present in infants with apparent life‐threatening events than in healthy infants (Wasilewska, Kaczmarski, et al. 2011; Wasilewska, Sienkiewicz‐Szlapka, et al. 2011).

In contrast to A1‐type β‐casein, little or no BCM‐7 is released from the digestion of A2‐type β‐casein (Ul Haq et al. 2015; Boutrou et al. 2013). Subsequently, consumption of milk free of A1‐type β‐casein (A1PF) may avoid BCM‐7‐related gastrointestinal and inflammatory effects. Indeed, there is increasing evidence showing that individuals, including toddlers and preschool children, who consume A1PF milk have improved gastrointestinal symptoms compared with those who consume conventional milk (containing both A1‐ and A2‐type β‐casein) (He et al. 2017; Brooke‐Taylor et al. 2017; Küllenberg de Gaudry et al. 2019; Sheng et al. 2019; Meng et al. 2023).

Thus, replacing conventional milk (i.e., the base ingredient of infant formula) with A1PF milk may support improved gastrointestinal comfort. To investigate this, we conducted this study to compare the effects of infant formula made with A1PF milk (A1PF infant formula) versus infant formula made with conventional milk (CON infant formula) on growth, gastrointestinal tolerance, and crying frequency and duration in Chinese mixed‐fed infants aged 3–4 months.

Key messages:

- Conventional milk can cause gastrointestinal discomfort and inflammation thought to be caused by A1 β‐casein through digestion to form β‐casomorphin‐7 (BCM‐7).
- Digestion of milk free of A1‐type β‐casein (A1PF) results in little to no BCM‐7, and consequently reduces gastrointestinal and inflammatory effects compared with conventional milk.
- This open‐label, randomized, controlled trial showed that compared with conventional infant formula, A1PF infant formula resulted in similar growth but fewer crying episodes in mixed (breastmilk and formula)‐fed infants.
- More parents/caregivers reported gastrointestinal symptom relief, less vomiting, improved digestion, and better stool characteristics with A1PF infant formula versus conventional infant formula.

## 2 Methods

### 2.1 Study Design and Treatments

This open‐label, randomized, controlled trial was conducted in Shanghai, China (14 July 2023–3 November 2023). The study duration was 56 days, and participants were allocated using a simple randomization method in SAS software, version 9.4 (SAS Institute Inc., Cary, NC, USA), by Adjuvant, the contract research organization, to the A1PF infant formula group or the CON infant formula group. The formulas were prepared and administered by parents/caregivers per the instructions on the label. The study was conducted in accordance with the ethical principles laid down in the Declaration of Helsinki, in compliance with Good Clinical Practice and all relevant regulatory requirements. The study was registered in ClinicalTrials.gov and was approved by the Shanghai Ethics Committee for Clinical Research (SECCR/2023‐51‐01, approval date 26 May 2023). All parents/caregivers of the participants provided written informed consent.

### 2.2 Participants

Healthy infants aged between 60 and 120 days, with a gestational age of 37–42 weeks and a birth weight of 2500–4500 g, and who were mixed‐fed according to the parents' choice (defined as regularly consuming both formula [> 400 mL/day at study entry and throughout the study] and breastmilk) were eligible to participate in the study. Infants with inborn malformations and hereditary and/or chronic and/or inborn diseases that could interfere with the study, evidence of feeding difficulties or intolerance/allergy to cows' milk, significant systemic disorders (e.g., cardiac, respiratory, endocrinological, haematologic, or gastrointestinal), acute infection or gastroenteritis at enrolment, or those participating in another clinical trial were excluded. Participants were recruited by a single institution (Shanghai Weierkang Pediatric Outpatient Department) and were contacted by telephone after a routine visit to the facility.

### 2.3 Study Assessments

Primary outcomes were anthropometric parameters (including body weight, length, body mass index [BMI], head circumference, and corresponding *Z*‐scores) and infant comfort (assessed by the Infant Gastrointestinal Symptom Questionnaire [IGSQ]; Riley et al. 2015 and a crying survey). The IGSQ comprises a 13‐item index score assessed across 5 domains: stooling, spitting up/vomiting, flatulence, crying, and fussiness. Items are scored on a scale of 1–5, with higher scores representing more severe symptoms; the total score can therefore range from 13 (no symptoms) to 65 (maximum discomfort). The crying survey comprised a simple sheet of a 24‐h day split into 15‐min time blocks. Parents/caregivers were instructed to shade in each time block in which their infant was crying. Secondary outcomes included eczema/dermatitis severity and quality of life (QoL) as evaluated by the Infants' Dermatitis QoL Index (IDQoL) (Lewis‐Jones et al. 2001; Cardiff University School of Medicine), an 11‐item survey (1 question for dermatitis severity and 10 questions for the life quality index) that asks participants to rate the severity of their symptoms. Dermatitis severity is scored on a scale of 0–4, with a higher score indicating higher severity; the 10 life quality index questions are scored on a scale of 0–3, with higher scores indicating greater impairment in quality of life. Other secondary outcomes included satisfaction with the study formula (via a survey) and adverse events (AEs), including respiratory issues, nappy rashes, and fever, which were recorded by the study investigator at each visit. The incidences of AEs and serious AEs were categorized using the Medical Dictionary for Regulatory Activities, version 22.0, n.d., by System Organ Class and Preferred Term.

**FIGURE 1:**
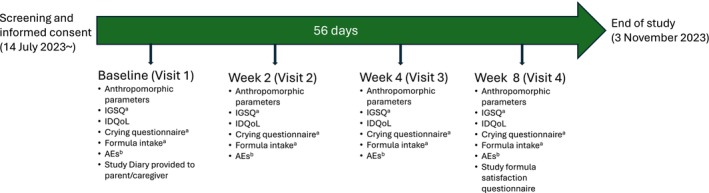
Study timeline. ^a^Recorded daily in subject diaries and reviewed by the investigator at each study visit. ^b^Recorded whenever an event occurred and reviewed by the investigator at each study visit. AEs, adverse events; IDQoL, Infants' Dermatitis Quality of Life Index; IGSQ, Infant Gastrointestinal Symptom Questionnaire.

Anthropometric measurements and the IDQoL were assessed at visit 1 (baseline), visit 2 (Week 2 ± 2 days), visit 3 (Week 4 ± 2 days) and visit 4 (Week 8 ± 2 days), and once‐daily assessments of the IGSQ, crying, and formula intake were recorded by the parents/caregivers throughout the study period using Study Diaries, which were reviewed at each visit (Figure 1) The satisfaction survey was conducted at the end of the study period (visit 4). Baseline measurements included sex, birth date, birth weight/length/head circumference, gestational age, method of delivery, maternal age, and medical history. Body weight, length, and head circumference were obtained from the hospital birth records or from the parent/caregiver.

### 2.4 Statistical Methods

By assuming a standard deviation (SD) of 6.0–7.0 g/day for the growth rate (Puccio et al. 2017) and a potential dropout rate of 30%, a sample size of 280 participants (140 per group) was calculated to provide at least 80% power to detect a 3 g/day difference in the weight growth rate from baseline to Day 56 of age (Week 8) between two groups with a two‐sided significance level of 0.05.

The intention‐to‐treat population comprised all eligible and enrolled infants and was analyzed to determine efficacy outcomes. The per‐protocol population consisted of all participants who completed the study and was used to evaluate satisfaction with the infant formulas. As this was an open‐label study, the participants, parents/caregivers, and investigators were not blinded to the study groups. An independent ethics committee provided guidance regarding strategies to minimize the risk of bias, and the statistician and assessors were blinded to the study groups.

Prior to summarizing and performing statistical comparisons, normality testing was conducted using a Shapiro–Wilk test. For non‐normal data, transformations or nonparametric tests were used. Continuous data were summarized by mean and SD or median and interquartile range, as appropriate. Categorical variables were summarized by frequency and percentage. Demographic and baseline characteristics were compared using a one‐way analysis of variance for continuous variables with normal distribution, Kruskal–Wallis test for non‐normal continuous variables, and a chi‐square or Fisher's exact test for categorical variables.

Anthropometric parameters and their corresponding *Z*‐scores were calculated according to the 2006 World Health Organization (WHO) Growth Standards (World Health Organization 2006). Least‐squares means were estimated and compared at each visit between the two groups. Baseline anthropometric differences between the groups were assessed using analysis of covariance (ANCOVA) with adjustments for age and sex. Between‐group comparisons at Weeks 2, 4, and 8 were assessed using ANCOVA with adjustments for baseline measurements, age, and sex. The individual IGSQ scores for each domain (stooling: questions 1 and 2, spitting up/vomiting: questions 3–6, crying: questions 7–9, fussiness: questions 10 and 11, and flatulence: questions 12 and 13) were summed, and the daily average over the past week at each visit was calculated. Composite IGSQ scores were compared between the groups using ANCOVA, adjusted for baseline composite IGSQ score, age, sex, and the average formula intake per day over the previous week for each visit. Crying episodes were summarized, and between‐group comparisons were assessed using ANCOVA with adjustments for age, sex, and the average formula intake per day over the previous week. The sum of the QoL scores was compared using a Kruskal–Wallis test. Formula satisfaction responses were summarized, and between‐group comparisons were conducted using a chi‐square or Fisher's exact test. The average formula intake (mL/day) and feeding duration (minutes/day) over the previous week at each visit were summarized, and between‐group comparisons were conducted using ANCOVA, adjusted for baseline age and sex. The frequency and intake of other foods were compared using a Kruskal–Wallis test. Fisher's exact test was used to compare the proportion of participants from each group with an AE or serious AE. Outcomes were also evaluated for the subgroup of infants with a family history of allergies.

Missing data after early withdrawal were not imputed for either analysis population. All statistical analyses were conducted using SAS software, version 9.4 (SAS Institute Inc.), with *p* < 0.05 considered statistically significant for two‐sided tests.

## 3 Results

### 3.1 Participants

**FIGURE 2:**
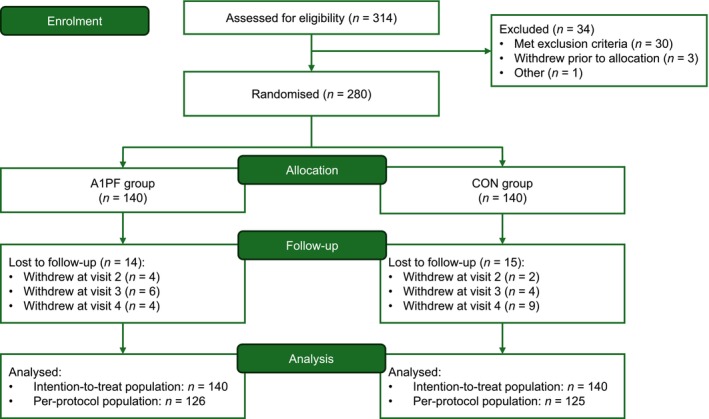
Participant flow diagram. Chinese mixed‐fed infants (aged 3–4 months) were enrolled. A1PF, infant formula made from milk free of A1‐type β‐casein; CON, infant formula made from conventional milk (containing both A1‐ and A2‐type β‐casein).

A total of 280 infants were included in this study and comprised the intention‐to‐treat population (Figure 2). The per‐protocol set included 126 and 125 participants in the A1PF and CON groups, respectively. Baseline demographics of the intention‐to‐treat population are shown in Table S1. Approximately half of the infants were female (A1PF group: 56.43%, CON group: 49.29%), and the mean (SD) age was 100.83 (15.01) days in the A1PF group and 99.28 (13.15) days in the CON group. No significant differences were observed between the groups regarding demographics or baseline characteristics.

### 3.2 Primary Outcomes: Anthropometric Measures, Gastrointestinal Symptoms, and Crying

The anthropometric measurements of the A1PF and CON groups were similar at baseline, and there were no significant between‐group differences in achieved weight, length, head circumference, BMI, or corresponding *Z*‐scores over time (Table 1).

**TABLE 1:** Anthropometric measurements over time (intention‐to‐treat population).

	A1PF group (*n* = 140)	CON group (*n* = 140)	Group difference	
			Difference of least‐squares means (95% CI)	*p*
Weight, g				
Baseline	6611.29 ± 776.18	6635.64 ± 670.84	−43.90 (−179.00, 91.20)	0.523
Week 2	7047.50 ± 780.69	7008.41 ± 683.39	47.83 (−16.06, 111.71)	0.142
Week 4	7369.85 ± 783.75	7334.5 5 ± 663.81	58.46 (−13.62, 130.54)	0.111
Week 8	7796.51 ± 760.79	7758.96 ± 676.98	55.19 (−37.48, 147.87)	0.242
Length, cm				
Baseline	62.22 ± 2.54	62.27 ± 2.24	−0.11 (−0.53, 0.30)	0.593
Week 2	63.63 ± 2.53	63.47 ± 2.20	0.13 (−0.05, 0.32)	0.163
Week 4	64.80 ± 2.60	64.63 ± 2.18	0.14 (−0.08, 0.36)	0.219
Week 8	66.48 ± 2.54	66.29 ± 2.19	−0.16 (−0.14, 0.46)	0.296
Head circumference, cm				
Baseline	40.38 ± 1.29	40.44 ± 1.30	−0.07 (−0.29, 0.15)	0.535
Week 2	40.93 ± 1.24	41.00 ± 1.26	−0.01 (−0.09, 0.07)	0.786
Week 4	41.49 ± 1.24	41.56 ± 1.20	−0.01 (−0.12, 0.10)	0.823
Week 8	42.31 ± 1.10	42.36 ± 1.11	−0.04 (−0.18, 0.09)	0.525
BMI, kg/m^2^				
Baseline	17.04 ± 1.32	17.09 ± 1.08	−0.06 (−0.34, 0.22)	0.667
Week 2	17.36 ± 1.07	17.37 ± 1.07	0.02 (−0.13, 0.18)	0.783
Week 4	17.51 ± 1.01	17.54 ± 0.99	0.03 (−0.13, 0.20)	0.696
Week 8	17.62 ± 1.04	17.64 ± 1.05	0.02 (−0.18, 0.22)	0.838
Weight‐for‐age *Z*‐score				
Baseline	0.41 ± 0.77	0.44 ± 0.66	−0.05 (−0.22, 0.11)	0.511
Week 2	0.58 ± 0.75	0.53 ± 0.64	0.05 (−0.02, 0.13)	0.158
Week 4	0.65 ± 0.74	0.60 ± 0.61	0.06 (−0.02, 0.15)	0.137
Week 8	0.60 ± 0.73	0.57 ± 0.62	0.06 (−0.04, 0.16)	0.268
Length‐for‐age *Z*‐score				
Baseline	0.47 ± 0.94	0.49 ± 0.75	−0.04 (−0.24, 0.16)	0.690
Week 2	0.60 ± 1.01	0.52 ± 0.76	0.06 (−0.03, 0.15)	0.170
Week 4	0.68 ± 1.02	0.58 ± 0.76	0.06 (−0.04, 0.17)	0.242
Week 8	0.61 ± 1.03	0.54 ± 0.81	0.06 (−0.07, 0.20)	0.360
Weight‐for‐length *Z*‐score				
Baseline	0.19 ± 0.87	0.20 ± 0.75	−0.03 (−0.22, 0.16)	0.744
Week 2	0.33 ± 0.66	0.31 ± 0.72	0.02 (−0.09, 0.13)	0.728
Week 4	0.39 ± 0.64	0.38 ± 0.66	0.03 (−0.09, 0.14)	0.624
Week 8	0.43 ± 0.67	0.41 ± 0.70	0.02 (−0.11, 0.16)	0.756
Head circumference‐for‐age *Z*‐score				
Baseline	0.08 ± 0.79	0.11 ± 0.79	−0.05 (−0.23, 0.13)	0.611
Week 2	0.10 ± 0.80	0.14 ± 0.79	−0.01 (−0.08, 0.06)	0.747
Week 4	0.19 ± 0.81	0.22 ± 0.75	−0.01 (−0.10, 0.08)	0.791
Week 8	0.19 ± 0.73	0.22 ± 0.71	−0.03 (−0.14, 0.07)	0.527
BMI‐for‐age *Z*‐score				
Baseline	0.21 ± 0.85	0.23 ± 0.71	−0.04 (−0.23, 0.14)	0.646
Week 2	0.34 ± 0.66	0.32 ± 0.69	0.02 (−0.08, 0.12)	0.688
Week 4	0.37 ± 0.64	0.37 ± 0.64	0.03 (−0.08, 0.14)	0.644
Week 8	0.35 ± 0.67	0.35 ± 0.69	0.02 (−0.11, 0.15)	0.749

*Note:* Data are shown as mean ± SD, unless otherwise stated. Group differences were calculated using an analysis of covariance, adjusted for age and sex. Post‐intervention analysis adjusted for baseline measurements.

Abbreviations: A1PF, infant formula made from milk free of A1‐type β‐casein; BMI, body mass index; CI, confidence interval; CON, infant formula made from conventional milk (containing both A1‐ and A2‐type β‐casein); SD, standard deviation.

**FIGURE 3:**
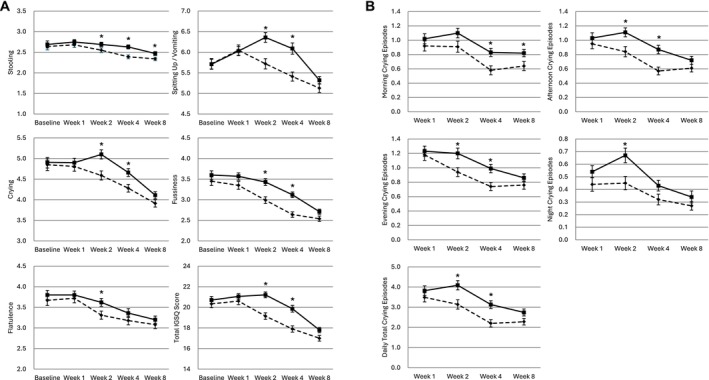
Change over time in gastrointestinal symptoms and crying episodes during the previous week (intention‐to‐treat population). Change over time in (A) daily infant gastrointestinal symptom questionnaire scores and (B) number of crying episodes of Chinese mixed‐fed infants (aged 3–4 months) during the previous week. Data are shown as mean scores with standard deviation. *Denotes timepoints with significant least‐squares mean differences between the groups (*p* < 0.05). The dashed line represents the A1PF group, and the solid line represents the CON group. A1PF, infant formula made from milk free of A1‐type β‐casein; CON, infant formula made from conventional milk (containing both A1‐ and A2‐type β‐casein); IGSQ, Infant Gastrointestinal Symptom Questionnaire.

Five‐domain IGSQ scores are shown in Figure 3A, and individual item scores, including between‐group differences, are shown in Table S2. Baseline individual item scores, five‐domain scores, and total IGSQ scores were similar between the groups. At Week 2, the A1PF group had significantly lower mean (SD) daily scores than the CON group for stooling (2.55 [0.67] vs. 2.69 [0.63], mean difference [95% confidence interval]: −0.14 [−0.27, −0.01]), spitting up/vomiting (5.72 [1.45] vs. 6.36 [1.40], difference: −0.66 [−0.98, −0.35]), crying (4.59 [1.29] vs. 5.10 [1.32], difference: −0.48 [−0.77, −0.20]), fussiness (2.99 [0.96] vs. 3.43 [0.98], difference: −0.38 [−0.59, −0.18]), and total IGSQ score (19.15 [3.74] vs. 21.21 [3.30], difference:−1.93 [−2.69, −1.17]) (all *p* < 0.05). These scores were also significantly lower in the A1PF group than the CON group at Week 4: stooling (2.39 [0.52] vs. 2.63 [0.55], difference: −0.23 [−0.35, −0.10]), spitting up/vomiting (5.41 [1.26] vs. 6.09 [1.58], difference: −0.65 [−0.99, −0.31]), crying (4.28 [1.07] vs. 4.66 [1.09], difference: −0.34 [−0.59, −0.08]), fussiness (2.64 [0.76] vs. 3.12 [0.78], difference: −0.42 [−0.60, −0.24]), and total IGSQ score (17.90 [3.49] vs. 19.86 [3.73], difference: −1.72 [−2.57, −0.88]) (all *p* < 0.05). The A1PF group had a significantly lower average flatulence score than the CON group at Week 2 (3.31 [1.15] vs. 3.62 [1.13], difference: −0.28 [−0.54, −0.01], *p* = 0.038), but not Week 4. In the individual item scores, the A1PF group had significantly lower scores for all items except hard stools and frequency of arching back at Week 2 (see Table S2). Similar results were observed regarding IGSQ scores among participants with a family history of allergy (see Table S3).

The average number of 15‐min crying periods per day is shown in Figure 3B and Table S4. During the first week, there were no significant differences in the number of crying episodes between the two groups. During Week 2, the A1PF group had a significantly lower mean (SD) number of crying episodes than the CON group in the afternoon (0.84 [0.83] vs. 1.11 [0.74], mean difference [95% confidence interval]: −0.27 [−0.45, −0.08], *p* = 0.005), evening (0.94 [0.72] vs. 1.20 [0.88], difference: −0.25 [−0.44, −0.06], *p* = 0.010), and night (0.45 [0.62] vs. 0.67 [0.69], difference: −0.20 [−0.36, −0.05], *p* = 0.010). The A1PF group also had significantly lower numbers of crying episodes than the CON group in the morning (0.58 [0.72] vs. 0.83 [0.66], difference: −0.24 [−0.40, −0.07], *p* = 0.007), afternoon (0.57 [0.62] vs. 0.87 [0.68], difference: −0.29 [−0.45, −0.13], *p* = 0.0003) and evening (0.74 [0.64] vs. 0.99 [0.67], difference: −0.25 [−0.41, −0.09], *p* = 0.003) of Week 4. The total mean (SD) number of daily crying episodes during Weeks 2 and 4 was significantly lower in the A1PF group than the CON group (3.14 [2.73] vs. 4.09 [2.70], difference: −0.91 [−1.55, −0.26] and 2.20 [2.10] vs. 3.13 [2.21], difference: −0.88 [−1.41, −0.35], respectively; both *p* < 0.05). The number of crying episodes tended to be lower in the A1PF group than the CON group at Week 8, but this was not statistically significant.

### 3.3 Secondary Outcomes: Dermatitis, Quality of Life, and Satisfaction With the Study Formulas

The IDQoL scores are shown in Table S5. Infants in the A1PF group had overall similar dermatitis severity and QoL scores to those in the CON group, which were consistent both at baseline and throughout the study. No differences between the groups in IDQoL scores were observed among participants with a family history of allergy (see Table S6). Infants in both groups had a similar amount of formula feeding and other food intake throughout the study (see Table S7).

Almost all parents/caregivers of infants in the A1PF group were satisfied with the formula (99.21%) (Table 2); this was significantly higher than the proportion satisfied with the formula in the CON group (91.20%), which included five parents/caregivers who were dissatisfied (*p* = 0.007). More parents/caregivers of infants in the A1PF group reported subjective relief of gastrointestinal symptoms than the CON group (81/126 [64.29%] vs. 54/125 [43.20%]; *p* = 0.001). Vomiting after formula feeding, digestion, and stool characteristics were also reported to be significantly improved in the A1PF group compared with the CON group (all *p* < 0.05).

**TABLE 2:** Satisfaction with the study formulas (per‐protocol population).

	A1PF group (*n* = 126)	CON group (*n* = 125)	*p*
1. Satisfaction with the study formula			
Satisfied	125 (99.21)	114 (91.20)	**0.007**
Neutral	1 (0.79)	6 (4.80)	
Dissatisfied	0 (0.00)	5 (4.00)	
Not sure	0 (0.00)	0 (0.00)	
2. Relief of the child's gastrointestinal symptoms after study formula intake	81 (64.29)	54 (43.20)	**0.001**
2a. Relieved gastrointestinal symptoms			
Vomiting after feeding	55 (43.65)	32 (25.60)	**0.003**
Flatulence	13 (10.32)	11 (8.80)	0.831
Burps	9 (7.14)	7 (5.60)	0.797
Digestion	42 (33.33)	19 (15.20)	**0.001**
Loose stool or other stool characteristics	40 (31.75)	23 (18.40)	**0.020**
Dietary protein intolerance	1 (0.79)	0 (0.00)	1.000
Unspecified	4 (3.17)	2 (1.60)	0.684
3. Relief of the child's skin symptoms after study formula intake	38 (30.16)	25 (20.00)	0.080
3a. Relieved skin symptoms			
Eczema	9 (7.14)	5 (4.00)	0.410
Dermatitis	21 (16.67)	16 (12.80)	0.477
Unspecified	8 (6.35)	4 (3.20)	0.376
4. Have intention of continuing to feed the child with the study formula			
Yes	114 (90.48)	103 (82.40)	0.148
No	2 (1.59)	6 (4.80)	
Not sure	10 (7.94)	16 (12.80)	

*Note:* Data shown as *n* (%). Group differences were evaluated using Fisher's exact test. Bold text denotes statistical significance (*p* < 0.05).

Abbreviations: A1PF, infant formula made from milk free of A1‐type β‐casein; CON, infant formula made from conventional milk (containing both A1‐ and A2‐type β‐casein).

### 3.4 Secondary Outcomes: Adverse Events

The frequency and type of AEs are shown in Table S8. Seventy‐three AEs were reported, and no significant differences were observed between the two groups. The most frequently reported AEs were fever (*n* = 14), upper respiratory infections and cough without other related symptoms (both *n* = 11), and eczema (*n* = 10). No serious AEs were reported, and no participants withdrew from the study because of an AE.

## 4 Discussion

This study compared the effects of A1PF infant formula and CON infant formula in infants who consumed both formula and breastmilk. Infants who consumed A1PF infant formula achieved the recommended growth according to the WHO standards (World Health Organization 2006) for infants in the relevant age groups. Moreover, infants in the A1PF group had reduced stooling, spitting up/vomiting after feeding, crying, and fussiness, compared with those in the CON group, after 4 weeks (mean difference in Week 4 IGSQ scores: −0.23, −0.65, −0.34, −0.42, respectively). This was reflected in the parent/caregiver satisfaction surveys, which stated that a higher percentage of infants in the A1PF group had improved relief of gastrointestinal symptoms (64.29% vs. 43.20%), including less vomiting after feeding (43.65% vs. 25.60%), improved digestion (33.33% vs. 15.20%), and improvement of loose stool or other stool characteristics (31.75% vs. 18.40%). Overall, the A1PF infant formula provided adequate nutrition and improved gastrointestinal symptoms compared with formula made with milk containing both A1‐ and A2‐type β‐casein in infants who consumed both formula and breastmilk.

Over the first 4 weeks, infants in the A1PF group had improved gastrointestinal symptoms, including a reduction in stooling, spitting up/vomiting, and fussiness, compared with the group who received CON infant formula. Additionally, the A1PF group had significantly fewer crying episodes than the CON group (Week 2 mean difference: −0.91 daily 15‐min crying periods; Week 4 difference: −0.88). A trend towards improved gastrointestinal symptoms and reduced crying was maintained at Week 8 in the A1PF group versus the CON group. The sample size of each group may have limited the ability to detect any significant differences at Week 8. These results in infants aged 3–4 months are consistent with studies including older children. For example, a previous study reported that toddlers (aged 12–36 months) who consumed A1PF milk had overall similar anthropometric parameters and improved gastrointestinal symptoms versus those who consumed conventional milk after 2 weeks (Meng et al. 2023). Similar findings have also been reported in preschool children (aged 5–6 years) who consumed A1PF milk (Sheng et al. 2019). The improved gastrointestinal symptoms and reduction in reported crying episodes were also reflected in the parent/caregiver satisfaction surveys, with more respondents in the A1PF group reporting relief of their infant's gastrointestinal symptoms and satisfaction with the formula than the CON group. These improvements may be explained by the lack of A1 β‐casein in the A1PF infant formula, thereby avoiding subsequent BCM‐7‐related effects. As BCM‐7 can cause inflammation and gastrointestinal discomfort (Woodford 2021; Sadler and Smith 2013), it is possible that infants who consumed CON infant formula experienced relatively worse gastrointestinal symptoms as a result of exposure to BCM‐7. Digestion of A1PF milk results in little to no BCM‐7, so it is likely that infants who consumed A1PF infant formula were exposed to far lower levels of BCM‐7. Further studies are warranted to confirm this hypothesis. Nevertheless, these observations suggest that infants who consumed A1PF infant formula had improved comfort compared with infants who consumed CON infant formula and support the use of A1PF milk as the base ingredient in infant formulas.

The infants who consumed infant formula made with A1PF milk had similar numbers and types of AEs as infants who consumed the CON infant formula. Following previous research (Puccio et al. 2017), all AEs were reviewed by investigators, and only those without other clear causes (e.g., illness or medication) were considered related to the study formula. Both formulas were generally well tolerated.

The main limitation of this study was its open‐label design, which carries an inherent risk of bias. However, an independent ethics committee provided guidance to mitigate these risks, including the use of blinded statisticians and assessors. Although mixed feeding may accurately reflect a common scenario for formula use, factors relating to breastmilk, including the mother's diet, could not be controlled and thus may have influenced the observed results. The small sample size may have contributed to the lack of significance regarding crying and comfort differences at Week 8. Future studies with a large population and a longer duration are needed to clarify effects on crying and gastrointestinal comfort beyond 4 weeks.

In conclusion, this study found that infants who consumed infant formula made with A1PF milk had appropriate growth and significantly reduced gastrointestinal discomfort compared to infants who consumed formula made with milk containing both A1‐ and A2‐type β‐casein.

## Author Contributions

**Jing Li:** conceptualization (equal), methodology (equal), writing – review and editing (equal). **Tingting Yang:** investigation (equal). **Xiaoyang Sheng:** conceptualization (equal), methodology (equal), writing – review and editing (equal).

## Ethics Statement

The study was conducted in accordance with the ethical principles laid down in the Declaration of Helsinki, in compliance with Good Clinical Practice and all relevant regulatory requirements. The study was registered in ClinicalTrials.gov and was approved by the Shanghai Ethics Committee for Clinical Research (SECCR/2023‐51‐01, approval date 2023/05/26). All parents/caregivers of the participants provided written informed consent.

## Conflicts of Interest

X.S. received speaker honoraria from The a2 Milk Company. J.L. and T.Y. have no conflicts to declare.

## Supporting information


Data S1.

## Data Availability

The data presented in this study are available from the corresponding author upon reasonable request.
