# Supplementary material for: Effect of Infant Formula Made With Milk Free of A1‐Type β‐Casein on Growth and Comfort: A Randomized Controlled Trial
Source: Food Sci Nutr. 2025 Jul 15;13(7):e70606. doi: 10.1002/fsn3.70606 (PMC12264312; doi:10.1002/fsn3.70606)
Supplement: Supplementary file 1 — Data S1. [file FSN3-13-e70606-s001.docx]

**Supporting Information**

**Supporting Information: Table 1.** Baseline demographics and assessments (intention-to-treat population)

|  | **A1PF group**  ***n* = 140** | **CON group**  ***n* = 140** | ***p-value*** |
| --- | --- | --- | --- |
| Age, days | 100.83 ± 15.01 | 99.28 ± 13.15 | 0.359 |
| Sex |  |  | 0.281 |
| Female | 79 (56.43) | 69 (49.29) |  |
| Male | 61 (43.57) | 71 (50.71) |  |
| Gestational age, day | 39.00 ± 0.97 | 39.05 ± 0.98 | 0.670 |
| Vaginal delivery | 89 (63.57) | 94 (67.14) | 0.616 |
| Birth weight, g | 3345.16 ± 349.51 | 3350.92 ± 362.97 | 0.892 |
| Birth length, cm | 49.87 ± 1.54 | 49.90 ± 1.76 | 0.871 |
| Head circumference at birth, cm | 34.18 ± 1.05 | 34.22 ± 1.00 | 0.757 |
| Apgar score | 10 (10–10) | 10 (10–10) | 0.665^†^ |
| Mother’s age at birth, years | 31.07 ± 4.26 | 31.19 ± 4.28 | 0.812 |
| Mother’s educational level |  |  | 0.427 |
| Senior high school/technical school | 13 (9.29) | 19 (13.57) |  |
| Bachelor’s degree | 110 (78.57) | 108 (77.14) |  |
| Master’s degree and above | 17 (12.14) | 13 (9.29) |  |
| Smoker(s) in the household | 34 (24.29) | 38 (27.14) | 0.682 |
| Pet(s) in the household | 35 (25.00) | 32 (22.86) | 0.780 |
| Primary caretaker |  |  | 0.723 |
| Mother | 137 (97.86) | 135 (96.43) |  |
| Grandmother | 3 (2.14) | 5 (3.57) |  |
| Number of house occupants | 4 (3–5) | 4 (3–4) | 0.809^†^ |
| Household size |  |  | 0.931 |
| <60 m^2^ | 23 (16.43) | 20 (14.29) |  |
| 60–<90 m^2^ | 65 (46.43) | 63 (45.00) |  |
| 90–≤120 m^2^ | 42 (30.00) | 46 (32.86) |  |
| >120 m^2^ | 10 (7.14) | 11 (7.86) |  |
| Monthly average household income^‡^ |  |  | 0.921 |
| <5000 RMB (<690 USD) | 2 (1.43) | 1 (0.71) |  |
| 5000–<10,000 RMB (690–<1380 USD) | 9 (6.43) | 10 (7.14) |  |
| 10,000–<15,000 RMB (1380–<2070 USD) | 36 (25.71) | 40 (28.57) |  |
| 15,000–≤30,000 (2070–≤4140 USD) | 37 (26.43) | 39 (27.86) |  |
| >30,000 RMB (>4140 USD) | 56 (40.00) | 50 (35.71) |  |
| Family medical history |  |  |  |
| Food allergy | 15 (10.71) | 18 (12.86) | 0.711 |
| Respiratory allergy | 51 (36.43) | 48 (34.29) | 0.803 |
| Contact allergy | 17 (12.14) | 21 (15.00) | 0.601 |
| Environmental allergy | 42 (30.00) | 46 (32.86) | 0.700 |
| Any above allergy | 83 (59.29) | 89 (63.57) | 0.539 |

*Note:* Data are shown as frequency (%), mean ± standard deviation, or median (interquartile range). Group differences were calculated using one-way analysis of variance for continuous variables and Fisher’s exact test for categorical variable, unless otherwise stated.

Abbreviations: A1PF, infant formula made from milk free of A1-type β-casein; CON, infant formula made from conventional milk (containing both A1- and A2-type β-casein); RMB, Chinese yuan; USD, United States dollar.

^†^Kruskal–Wallis test.

^‡^Approximate USD amounts are based on historical exchange rates per https://www.xe.com/ (1 RMB = 0.138 USD).

**Supporting Information: Table 2.** Average daily infant gastrointestinal symptom questionnaire scores over the past week (intention-to-treat population)

|  | **A1PF group**  ***n* = 140** | **CON group**  ***n* = 140** | **Group difference (A1PF versus CON)** | |
| --- | --- | --- | --- | --- |
|  |  |  | **Difference of least-squares means (95% CI)** | ***p*-value** |
| **Stooling** | | | | |
| Baseline | 2.64 ± 0.97 | 2.69 ± 1.00 | −0.02 (−0.25, 0.21) | 0.880 |
| Week 1 | 2.68 ± 0.73 | 2.75 ± 0.66 | −0.06 (−0.19, 0.07) | 0.338 |
| Week 2 | 2.55 ± 0.67 | 2.69 ± 0.63 | −0.14 (−0.27, −0.01) | **0.035** |
| Week 4 | 2.39 ± 0.52 | 2.63 ± 0.55 | −0.23 (−0.35, −0.10) | **0.0004** |
| Week 8 | 2.34 ± 0.45 | 2.47 ± 0.51 | −0.12 (−0.23, -0.004) | **0.043** |
| 1. Hard stools | | | | |
| Baseline | 1 (1–2) [1–4] | 1 (1–2) [1, 5] | / | 0.989 |
| Week 1 | 1.29 (1–1.71) [1–4.57] | 1.29 (1–1.71) [1–3] | / | 0.410 |
| Week 2 | 1.14 (1–1.57) [1–4.14] | 1.29 (1–1.57) [1–3.14] | / | 0.051 |
| Week 4 | 1 (1–1.43) [1–4] | 1.29 (1–1.57) [1–2.71] | / | **0.001** |
| Week 8 | 1 (1–1.29) [1–2.71] | 1.29 (1–1.43) [1–3] | / | **0.011** |
| 2. Difficulty in passing stool | | | | |
| Baseline | 1 (1–1) [1–3] | 1 (1–1) [1–3] | / | 0.222 |
| Week 1 | 1.14 (1–1.43) [1–2.57] | 1.14 (1–1.5) [1–2.43] | / | 0.068 |
| Week 2 | 1.14 (1–1.29) [1–2.71] | 1.14 (1–1.43) [1–2.57] | / | **0.003** |
| Week 4 | 1 (1–1.14) [1–2.29] | 1.29 (1–1.43) [1–2.29] | / | **<0.0001** |
| Week 8 | 1 (1–1.14) [1–2] | 1.14 (1–1.29) [1–2.57] | / | **0.002** |
| **Spitting up / vomiting** | | | | |
| Baseline | 5.72 ± 1.51 | 5.71 ± 1.44 | 0.05 (−0.30, 0.39) | 0.776 |
| Week 1 | 6.05 ± 1.49 | 6.03 ± 1.33 | −0.01 (−0.31, 0.30) | 0.959 |
| Week 2 | 5.72 ± 1.45 | 6.36 ± 1.40 | −0.66 (−0.98, −0.35) | **<0.0001** |
| Week 4 | 5.41 ± 1.26 | 6.09 ± 1.58 | −0.65 (−0.99, −0.31) | **0.0002** |
| Week 8 | 5.13 ± 1.19 | 5.32 ± 1.01 | −0.19 (−0.46, 0.07) | 0.152 |
| 3. Frequency of spit up | | | | |
| Baseline | 2 (1–2) [1–4] | 2 (1–2) [1–4] | / | 0.704 |
| Week 1 | 2 (1.57–2.43) [1–5] | 2.14 (1.86–2.43) [1–3.86] | / | 0.164 |
| Week 2 | 1.93 (1.29–2.29) [1–5] | 2.29 (2–2.57) [1–3.29] | / | **<0.0001** |
| Week 4 | 1.71 (1.14–2.14) [1–3.57] | 2 (1.71–2.43) [1–4] | / | **<0.0001** |
| Week 8 | 1.64 (1–2) [1–4] | 1.71 (1.43–2) [1–3.14] | / | **0.037** |
| 4. Volume of milk spit up | | | | |
| Baseline | 1 (1–1) [1–3] | 1 (1–1) [1–3] | / | 0.680 |
| Week 1 | 1.14 (1–1.43) [1–5] | 1.14 (1–1.43) [1–2.57] | / | 0.831 |
| Week 2 | 1 (1–1.29) [1–3] | 1.29 (1–1.43) [1–2.57] | / | **0.004** |
| Week 4 | 1 (1–1.29) [1–2.29] | 1.14 (1–1.43) [1–3] | / | **0.002** |
| Week 8 | 1 (1–1.14) [1–2.43] | 1 (1–1.29) [1–2.14] | / | 0.133 |
| 5. Discomfort when spitting up | | | | |
| Baseline | 1 (1–2) [1–3] | 1 (1–2) [1–3] | / | 0.671 |
| Week 1 | 1.43 (1–2) [1–3.14] | 1.43 (1.14–1.86) [1–3.29] | / | 0.808 |
| Week 2 | 1.29 (1–1.86) [1–3] | 1.71 (1.29–2.07) [1–4.57] | / | **<0.0001** |
| Week 4 | 1.29 (1–1.71) [1–3] | 1.57 (1.14–2) [1–5] | / | **<0.0001** |
| Week 8 | 1 (1–1.43) [1–3] | 1.29 (1–1.57) [1–2.71] | / | **0.036** |
| 6. Frequency of arching back | | | | |
| Baseline | 1 (1–1) [1–3] | 1 (1–1) [1–3] | / | 0.689 |
| Week 1 | 1 (1–1.14) [1–2.57] | 1 (1–1) [1–3.71] | / | 0.105 |
| Week 2 | 1 (1–1) [1–2.86] | 1 (1–1) [1–3.14] | / | 0.983 |
| Week 4 | 1 (1–1) [1–2.71] | 1 (1–1) [1–4.86] | / | 0.296 |
| Week 8 | 1 (1–1) [1–3] | 1 (1–1) [1–2.14] | / | 0.207 |
| **Crying** | | | | |
| Baseline | 4.85 ± 1.65 | 4.91 ± 1.33 | −0.05 (−0.40, 0.31) | 0.790 |
| Week 1 | 4.81 ± 1.38 | 4.90 ± 1.24 | −0.06 (−0.34, 0.22) | 0.683 |
| Week 2 | 4.59 ± 1.29 | 5.10 ± 1.32 | −0.48 (−0.77, −0.20) | **0.001** |
| Week 4 | 4.28 ± 1.07 | 4.66 ± 1.09 | −0.34 (−0.59, −0.08) | **0.011** |
| Week 8 | 3.91 ± 1.00 | 4.11 ± 0.94 | −0.18 (−0.42, 0.05) | 0.129 |
| 7. Total crying time | | | | |
| Baseline | 2 (1.5–2) [1–5] | 2 (2–2) [1–4] | / | 0.888 |
| Week 1 | 1.86 (1.43–2.14) [1–4] | 1.79 (1.57–2.14) [1–3.86] | / | 0.687 |
| Week 2 | 1.86 (1.29–2) [1–4] | 2 (1.57–2.29) [1–4] | / | **0.008** |
| Week 4 | 1.86 (1.29–2) [1–4] | 1.86 (1.57–2.14) [1–3.86] | / | 0.070 |
| Week 8 | 1.43 (1–1.86) [1–3.14] | 1.57 (1.29–2) [1–3.71] | / | 0.058 |
| 8. Could not soothe crying | | | | |
| Baseline | 1 (1–2) [1–5] | 1 (1–2) [1–4] | / | 0.127 |
| Week 1 | 1.14 (1–1.57) [1–3.29] | 1.29 (1–1.43) [1–3.14] | / | 0.500 |
| Week 2 | 1 (1–1.43) [1–3] | 1.29 (1–1.64) [1–2.57] | / | **0.002** |
| Week 4 | 1 (1–1.29) [1–3] | 1.14 (1–1.43) [1–2.29] | / | **0.044** |
| Week 8 | 1 (1, 1.14) [1–3] | 1 (1–1.29) [1–2.57] | / | **0.024** |
| 9. Crying after feeding | | | | |
| Baseline | 1 (1–2) [1–4] | 1 (1–2) [1–4] | / | 0.982 |
| Week 1 | 1.43 (1, 2) [1–3.86] | 1.71 (1.14–2.29) [1–4] | / | **0.008** |
| Week 2 | 1.29 (1–1.86) [1–3.57] | 1.71 (1.14–2.14) [1–3.86] | / | **0.001** |
| Week 4 | 1.14 (1–1.57) [1–3] | 1.43 (1–1.71) [1–4.86] | / | **0.001** |
| Week 8 | 1 (1–1.43) [1–3.57] | 1.14 (1–1.43) [1–2.71] | / | **0.040** |
| **Fussiness** | | | | |
| Baseline | 3.45 ± 1.24 | 3.60 ± 1.19 | −0.13 (−0.42, 0.15) | 0.357 |
| Week 1 | 3.35 ± 1.13 | 3.57 ± 0.97 | −0.14 (−0.37, 0.08) | 0.200 |
| Week 2 | 2.99 ± 0.96 | 3.43 ± 0.98 | −0.38 (−0.59, −0.18) | **0.0003** |
| Week 4 | 2.64 ± 0.76 | 3.12 ± 0.78 | −0.42 (−0.60, −0.24) | **<0.0001** |
| Week 8 | 2.54 ± 0.72 | 2.71 ± 0.68 | −0.12 (−0.28, 0.04) | 0.140 |
| 10. Frequency of fussiness | | | | |
| Baseline | 2 (2–3) [1–5] | 2 (2–3) [1–4] | / | 0.286 |
| Week 1 | 1.75 (1–2.25) [1–3] | 2 (1.75–2.5) [1–3.75] | / | **<0.0001** |
| Week 2 | 1.57 (1.14–2.14) [1–3] | 2 (1.57–2.57) [1–3.57] | / | **<0.0001** |
| Week 4 | 1.43 (1–1.71) [1–3] | 1.86 (1.57–2.29) [1–4.57] | / | **<0.0001** |
| Week 8 | 1.29 (1–1.57) [1–3.57] | 1.43 (1.14–1.86) [1–3.29] | / | **0.001** |
| 11. Could not soothe fussiness | | | | |
| Baseline | 1 (1–1) [1–5] | 1 (1–2) [1–4] | / | 0.252 |
| Week 1 | 1.2 (1–1.6) [1–3] | 1.4 (1–1.7) [1–2.6] | / | **0.010** |
| Week 2 | 1.14 (1–1.43) [1–3] | 1.29 (1–1.71) [1–2.57] | / | **0.002** |
| Week 4 | 1 (1–1.14) [1–3] | 1.14 (1–1.29) [1–2.57] | / | **0.0003** |
| Week 8 | 1 (1–1.14) [1–2.86] | 1 (1–1.14) [1–2.29] | / | 0.527 |
| **Flatulence** | | | | |
| Baseline | 3.67 ± 1.48 | 3.80 ± 1.30 | −0.10 (−0.43, 0.23) | 0.546 |
| Week 1 | 3.72 ± 1.28 | 3.80 ± 1.12 | −0.01 (−0.27, 0.24) | 0.909 |
| Week 2 | 3.31 ± 1.15 | 3.62 ± 1.13 | −0.28 (−0.54, −0.01) | **0.038** |
| Week 4 | 3.18 ± 1.20 | 3.36 ± 1.27 | −0.09 (−0.38, 0.19) | 0.521 |
| Week 8 | 3.08 ± 1.08 | 3.20 ± 0.99 | −0.07 (−0.31, 0.18) | 0.589 |
| 12. Frequency of gassiness | | | | |
| Baseline | 2 (1–3) [1–5] | 2 (2–3) [1–5] | / | 0.508 |
| Week 1 | 2 (1.33–2.33) [1–5] | 2 (1.58–2.5) [1–5] | / | 0.065 |
| Week 2 | 1.71 (1.29–2.29) [1–5] | 2 (1.43–2.57) [1–5] | / | **0.020** |
| Week 4 | 1.71 (1–2.29) [1–5] | 1.79 (1.29–2.29) [1–5] | / | 0.246 |
| Week 8 | 1.57 (1–2) [1–5] | 1.71 (1.29–2.14) [1–5] | / | 0.189 |
| 13. Discomfort due to gas | | | | |
| Baseline | 1 (1–2) [1–3] | 1 (1–2) [1–3] | / | 0.162 |
| Week 1 | 1.29 (1–1.57) [1–3] | 1.43 (1.14–1.93) [1–3.14] | / | **0.008** |
| Week 2 | 1.29 (1–1.57) [1–3.14] | 1.43 (1.14–1.93) [1–3.14] | / | **0.004** |
| Week 4 | 1.14 (1–1.57) [1–3] | 1.29 (1–1.71) [1–5] | / | **0.013** |
| Week 8 | 1.14 (1–1.43) [1–2.86] | 1.29 (1–1.57) [1–2.43] | / | **0.048** |
| **Total IGSQ score** | | | | |
| Baseline | 20.34 ± 4.44 | 20.71 ± 3.95 | −0.25 (−1.23, 0.73) | 0.615 |
| Week 1 | 20.61 ± 4.03 | 21.06 ± 3.25 | −0.28 (−1.05, 0.49) | 0.473 |
| Week 2 | 19.15 ± 3.74 | 21.21 ± 3.30 | −1.93 (−2.69, −1.17) | **<0.0001** |
| Week 4 | 17.90 ± 3.49 | 19.86 ± 3.73 | −1.72 (−2.57, −0.88) | **<0.0001** |
| Week 8 | 17.00 ± 3.11 | 17.80 ± 2.78 | −0.68 (−1.37, 0.02) | 0.058 |

*Note:* Data are shown as mean ± standard deviation or median (interquartile range) [range]. Baseline data were based on 24-hour recall of symptoms of the previous day. Baseline group difference was evaluated using analysis of covariance, adjusted for age and sex. Post-intervention group differences were calculated using analysis of covariance, adjusted for age, sex, and average daily formula intake in the past week. Post-intervention analyses were also adjusted for baseline scores. Bold text denotes statistical significance (*p* < 0.05).

Abbreviations: A1PF, infant formula made from milk free of A1-type β-casein; CI, confidence interval; CON, infant formula made from conventional milk (containing both A1- and A2-type β-casein); IGSQ, Infant Gastrointestinal Symptom Questionnaire.

**Supporting Information: Table 3.** Average daily infant gastrointestinal symptom questionnaire scores over the previous week among participants with family history of allergy (intention-to-treat population)

|  | **A1PF group**  ***n* = 140** | **CON group**  ***n* = 140** | **Group difference (A1PF versus CON)** | |
| --- | --- | --- | --- | --- |
|  |  |  | **Difference of least-squares means (95% CI)** | ***p*-value** |
| **Stooling** | | | | |
| Baseline | 2.76 ± 1.08 | 2.62 ± 0.99 | −0.17 (−0.60, 0.27) | 0.449 |
| Week 1 | 2.74 ± 0.77 | 2.75 ± 0.70 | −0.07 (−0.24, 0.10) | 0.405 |
| Week 2 | 2.60 ± 0.67 | 2.70 ± 0.66 | −0.17 (−0.33, 0.002) | 0.053 |
| Week 4 | 2.40 ± 0.50 | 2.71 ± 0.60 | −0.30 (−0.47, −0.14) | **0.001** |
| Week 8 | 2.39 ± 0.48 | 2.55 ± 0.59 | −0.14 (−0.31, 0.02) | 0.084 |
| 1. Hard stools | | | | |
| Baseline | 1 (1–2) [1–4] | 1 (1–2) [1–5] | / | 0.197 |
| Week 1 | 1.29 (1–1.86) [1–3.57] | 1.29 (1–1.71) [1–3] | / | 0.593 |
| Week 2 | 1.29 (1–1.57) [1–2.57] | 1.29 (1–1.57) [1–3.14] | / | 0.626 |
| Week 4 | 1.14 (1–1.43) [1–2.29] | 1.29 (1–1.71) [1–2.71] | / | **0.008** |
| Week 8 | 1.14 (1–1.43) [1–2.14] | 1.29 (1–1.43) [1–3] | / | 0.147 |
| 2. Difficulty in passing stool | | | | |
| Baseline | 1 (1–1) [1–3] | 1 (1–1) [1–3] | / | 0.534 |
| Week 1 | 1.14 (1–1.43) [1–2.57] | 1.14 (1–1.57) [1–2.43] | / | 0.102 |
| Week 2 | 1 (1–1.29) [1–2.71] | 1.29 (1–1.43) [1–2.29] | / | **0.005** |
| Week 4 | 1 (1–1.14) [1–2.29] | 1.29 (1.14–1.43) [1–2.29] | / | **<0.0001** |
| Week 8 | 1 (1–1.14) [1–2] | 1.14 (1–1.29) [1–2.57] | / | **0.002** |
| **Spitting up / vomiting** | | | | |
| Baseline | 5.60 ± 1.39 | 5.80 ± 1.44 | −0.17 (−0.60, 0.27) | 0.449 |
| Week 1 | 5.79 ± 1.23 | 6.03 ± 1.42 | −0.20 (−0.58, 0.17) | 0.283 |
| Week 2 | 5.57 ± 1.40 | 6.39 ± 1.51 | −0.75 (−1.18, −0.32) | **0.001** |
| Week 4 | 5.36 ± 1.35 | 6.17 ± 1.68 | −0.71 (−1.19, −0.22) | **0.005** |
| Week 8 | 5.17 ± 1.22 | 5.24 ± 0.97 | −0.04 (−0.39, 0.31) | 0.807 |
| 3. Frequency of spit up | | | | |
| Baseline | 2 (1–2) [1–4] | 2 (2–2) [1–4] | / | 0.500 |
| Week 1 | 2 (1.57–2.43) [1–5] | 2.14 (1.71–2.43) [1–3.86] | / | 0.142 |
| Week 2 | 1.86 (1.29–2.14) [1–5] | 2.29 (1.86–2.57) [1–3.29] | / | **<0.0001** |
| Week 4 | 1.86 (1.14–2.14) [1–3.57] | 2 (1.71–2.43) [1–4] | / | **0.001** |
| Week 8 | 1.57 (1–2) [1–4] | 1.71 (1.29–2) [1–3] | / | 0.219 |
| 4. Volume of milk spit up | | | | |
| Baseline | 1 (1–1) [1–2] | 1 (1–1) [1–3] | / | 0.149 |
| Week 1 | 1 (1–1.29) [1–2.86] | 1.14 (1–1.43) [1–2.57] | / | 0.115 |
| Week 2 | 1 (1–1.29) [1–2.57] | 1.29 (1–1.57) [1–2.43] | / | **0.001** |
| Week 4 | 1 (1–1.14) [1–2.29] | 1.14 (1–1.43) [1–3] | / | **0.0002** |
| Week 8 | 1 (1–1.14) [1–2.14] | 1 (1–1.29) [1–2] | / | 0.155 |
| 5. Discomfort when spitting up | | | | |
| Baseline | 1 (1–2) [1–3] | 1 (1–2) [1–3] | / | 0.378 |
| Week 1 | 1.43 (1–1.86) [1–3] | 1.43 (1.14–1.86) [1–3.29] | / | 0.544 |
| Week 2 | 1.29 (1–1.86) [1–3] | 1.57 (1.29–2) [1–4.57] | / | **<0.0001** |
| Week 4 | 1.14 (1–1.57) [1–3] | 1.57 (1.14–2) [1–5] | / | **0.0004** |
| Week 8 | 1 (1–1.57) [1–3] | 1.14 (1–1.57) [1–2.57] | / | 0.381 |
| 6. Frequency of arching back | | | | |
| Baseline | 1 (1–1) [1–3] | 1 (1–1) [1–3] | / | 0.721 |
| Week 1 | 1 (1–1) [1–2.14] | 1 (1–1) [1–3.71] | / | 0.961 |
| Week 2 | 1 (1–1) [1–2.86] | 1 (1–1) [1–3.14] | / | 0.329 |
| Week 4 | 1 (1–1) [1–2.71] | 1 (1–1) [1–4.86] | / | 0.093 |
| Week 8 | 1 (1–1) [1–3] | 1 (1–1) [1–1.86] | / | 0.241 |
| **Crying** | | | | |
| Baseline | 4.70 ± 1.68 | 4.91 ± 1.40 | −0.16 (−0.63, 0.31) | 0.503 |
| Week 1 | 4.58 ± 1.18 | 4.90 ± 1.30 | −0.24 (−0.59, 0.11) | 0.182 |
| Week 2 | 4.49 ± 1.28 | 5.21 ± 1.36 | −0.61 (−0.99, −0.23) | **0.002** |
| Week 4 | 4.29 ± 1.14 | 4.65 ± 1.18 | −0.25 (−0.61, 0.11) | 0.167 |
| Week 8 | 3.89 ± 1.04 | 4.01 ± 0.90 | −0.07 (−0.37, 0.24) | 0.668 |
| 7. Total crying time | | | | |
| Baseline | 2 (1–2) [1–5] | 2 (2–2) [1–3] | / | 0.709 |
| Week 1 | 1.71 (1.43–2) [1–4] | 1.71 (1.43–2.14) [1–3.29] | / | 0.498 |
| Week 2 | 1.86 (1.29–2) [1–4] | 2 (1.71–2.29) [1–3.14] | / | **0.0004** |
| Week 4 | 1.71 (1.29–2) [1–4] | 1.86 (1.57–2.14) [1–3.29] | / | 0.091 |
| Week 8 | 1.43 (1–1.86) [1–3.14] | 1.57 (1.29–1.86) [1–3.71] | / | 0.258 |
| 8. Unable to soothe crying | | | | |
| Baseline | 1 (1–2) [1–5] | 1 (1–2) [1–4] | / | 0.239 |
| Week 1 | 1 (1–1.29) [1–3] | 1.14 (1–1.43) [1–3.14] | / | 0.118 |
| Week 2 | 1 (1–1.29) [1–3] | 1.29 (1–1.71) [1–2.57] | / | **0.001** |
| Week 4 | 1 (1–1.29) [1–3] | 1.14 (1–1.29) [1–2.29] | / | 0.228 |
| Week 8 | 1 (1–1) [1–3] | 1 (1–1.14) [1–2.57] | / | **0.019** |
| 9. Crying after feeding | | | | |
| Baseline | 1 (1–2) [1–4] | 1 (1–2) [1–3] | / | 0.251 |
| Week 1 | 1.43 (1–2) [1–3] | 1.71 (1.29–2.29) [1–4] | / | **0.008** |
| Week 2 | 1.29 (1–1.86) [1–3] | 1.71 (1.14–2.14) [1–3.86] | / | **0.003** |
| Week 4 | 1.14 (1–1.57) [1–3] | 1.43 (1–1.71) [1–4.86] | / | **0.019** |
| Week 8 | 1 (1–1.43) [1–3.57] | 1.14 (1–1.43) [1–2.43] | / | 0.281 |
| **Fussiness** | | | | |
| Baseline | 3.52 ± 1.33 | 3.70 ± 1.20 | −0.11 (−0.49, 0.28) | 0.579 |
| Week 1 | 3.27 ± 1.17 | 3.58 ± 1.03 | −0.18 (−0.47, 0.11) | 0.216 |
| Week 2 | 3.01 ± 1.03 | 3.56 ± 1.07 | −0.47 (−0.75, −0.19) | **0.001** |
| Week 4 | 2.67 ± 0.82 | 3.16 ± 0.85 | −0.38 (−0.64, −0.13) | **0.003** |
| Week 8 | 2.58 ± 0.76 | 2.69 ± 0.62 | −0.05 (−0.26, 0.16) | 0.630 |
| 10. Frequency of fussiness | | | | |
| Baseline | 2 (2–3) [1–5] | 2 (2–3) [1–4] | / | 0.214 |
| Week 1 | 1.75 (1.25–2.25) [1–3] | 2.25 (1.75–2.75) [1–3.75] | / | **0.0004** |
| Week 2 | 1.57 (1.14–2) [1–3] | 2 (1.57–2.57) [1–3.57] | / | **0.0002** |
| Week 4 | 1.43 (1–1.71) [1–3] | 1.86 (1.43–2.14) [1–4.57] | / | **<0.0001** |
| Week 8 | 1.29 (1–1.57) [1–3.57] | 1.43 (1.14–1.86) [1–2.86] | / | **0.016** |
| 11. Unable to soothe fussiness | | | | |
| Baseline | 1 (1–1) [1–5] | 1 (1–2) [1–4] | / | 0.295 |
| Week 1 | 1.2 (1–1.6) [1–3] | 1.4 (1–1.8) [1–2.6] | / | **0.009** |
| Week 2 | 1.14 (1–1.29) [1–3] | 1.29 (1–1.71) [1–2.57] | / | **0.001** |
| Week 4 | 1 (1–1.14) [1–3] | 1.14 (1–1.29) [1–2.57] | / | **0.003** |
| Week 8 | 1 (1–1.14) [1–2.86] | 1 (1–1.14) [1–2.29] | / | 0.449 |
| **Flatulence** | | | | |
| Baseline | 3.76 ± 1.51 | 3.74 ± 1.31 | 0.08 (−0.36, 0.51) | 0.728 |
| Week 1 | 3.66 ± 1.32 | 3.82 ± 1.17 | −0.12 (−0.47, 0.22) | 0.485 |
| Week 2 | 3.35 ± 1.24 | 3.69 ± 1.13 | −0.32 (−0.66, 0.03) | 0.075 |
| Week 4 | 3.12 ± 1.21 | 3.39 ± 1.33 | −0.16 (−0.56, 0.23) | 0.406 |
| Week 8 | 2.92 ± 1.01 | 3.19 ± 1.01 | −0.19 (−0.51, 0.13) | 0.237 |
| 12. Frequency of gassiness | | | | |
| Baseline | 2 (1–3) [1–5] | 2 (2–3) [1–5] | / | 0.959 |
| Week 1 | 2 (1.33–2.33) [1–5] | 2 (1.67–2.5) [1–5] | / | 0.070 |
| Week 2 | 1.86 (1.29–2.29) [1–5] | 2.14 (1.43–2.57) [1–5] | / | **0.021** |
| Week 4 | 1.57 (1–2.29) [1–5] | 1.86 (1.29–2.29) [1–5] | / | 0.170 |
| Week 8 | 1.57 (1–2) [1–5] | 1.71 (1.29–2) [1–5] | / | **0.045** |
| 13. Discomfort due to gas | | | | |
| Baseline | 1 (1–2) [1–3] | 1 (1–2) [1–3] | / | 0.668 |
| Week 1 | 1.29 (1–1.57) [1–3] | 1.43 (1.14–1.86) [1–3.14] | / | **0.023** |
| Week 2 | 1.29 (1–1.57) [1–3.14] | 1.43 (1.14–2) [1–3.14] | / | **0.025** |
| Week 4 | 1 (1–1.57) [1–3] | 1.29 (1–1.71) [1–5] | / | **0.017** |
| Week 8 | 1.14 (1–1.43) [1–2.57] | 1.29 (1–1.57) [1–2.29] | / | 0.079 |
| **Total IGSQ score** | | | | |
| Baseline | 20.34 ± 4.56 | 20.76 ± 3.98 | −0.16 (−1.46, 1.13) | 0.804 |
| Week 1 | 20.05 ± 3.77 | 21.08 ± 3.65 | −0.79 (−1.80, 0.21) | 0.122 |
| Week 2 | 19.02 ± 3.99 | 21.55 ± 3.55 | −2.30 (−3.34, −1.26) | **<0.0001** |
| Week 4 | 17.84 ± 3.91 | 20.09 ± 4.18 | −1.80 (−3.04, −0.55) | **0.005** |
| Week 8 | 16.94 ± 3.35 | 17.68 ± 2.73 | −0.50 (−1.44, 0.43) | 0.289 |

*Note:* Data are shown as mean ± standard deviation or median (interquartile range) [range]. Baseline data were based on 24-hour recall of symptoms of the previous day. Baseline group difference was evaluated using analysis of covariance, adjusted for age and sex. Post-intervention group differences were calculated using analysis of covariance, adjusted for age, sex, and average daily formula intake in the past week. Post-intervention analyses were also adjusted for baseline scores. Bold text denotes statistical significance (*p* < 0.05).

Abbreviations: A1PF, infant formula made from milk free of A1-type β-casein; CI, confidence interval; CON, infant formula made from conventional milk (containing both A1- and A2-type β-casein); IGSQ, Infant Gastrointestinal Symptom Questionnaire.

**Supporting Information: Table 4.** Number of 15-minute crying periods per day in the past week (intention-to-treat population)

|  | **A1PF group**  ***n* = 140** | **CON group**  ***n* = 140** | **Group difference (A1PF versus CON)** | |
| --- | --- | --- | --- | --- |
|  |  |  | **Difference of least-squares means (95% CI)** | ***p*-value** |
| Morning, number of 15-minute periods | | | | |
| Week 1 | 0.92 ± 0.83 | 1.02 ± 0.84 | −0.10 (−0.30, 0.10) | 0.327 |
| Week 2 | 0.91 ± 0.90 | 1.10 ± 0.78 | −0.18 (−0.38, 0.02) | 0.070 |
| Week 4 | 0.58 ± 0.72 | 0.83 ± 0.66 | −0.24 (−0.40, −0.07) | **0.007** |
| Week 8 | 0.64 ± 0.70 | 0.82 ± 0.56 | −0.17 (−0.33, −0.01) | **0.037** |
| Afternoon, number of 15-minute periods | | | | |
| Week 1 | 0.95 ± 0.80 | 1.03 ± 0.88 | −0.09 (−0.29, 0.11) | 0.368 |
| Week 2 | 0.84 ± 0.83 | 1.11 ± 0.74 | −0.27 (−0.45, −0.08) | **0.005** |
| Week 4 | 0.57 ± 0.62 | 0.87 ± 0.68 | −0.29 (−0.45, −0.13) | **0.0003** |
| Week 8 | 0.61 ± 0.60 | 0.72 ± 0.62 | −0.11 (−0.27, 0.04) | 0.151 |
| Evening, number of 15-minute periods | | | | |
| Week 1 | 1.18 ± 0.91 | 1.23 ± 0.84 | −0.04 (−0.25, 0.16) | 0.681 |
| Week 2 | 0.94 ± 0.72 | 1.20 ± 0.88 | −0.25 (−0.44, −0.06) | **0.010** |
| Week 4 | 0.74 ± 0.64 | 0.99 ± 0.67 | −0.25 (−0.41, −0.09) | **0.003** |
| Week 8 | 0.76 ± 0.64 | 0.86 ± 0.63 | −0.10 (−0.26, 0.06) | 0.208 |
| Night, number of 15-minute periods | | | | |
| Week 1 | 0.44 ± 0.64 | 0.54 ± 0.57 | −0.10 (−0.24, 0.04) | 0.168 |
| Week 2 | 0.45 ± 0.62 | 0.67 ± 0.69 | −0.20 (−0.36, −0.05) | **0.010** |
| Week 4 | 0.32 ± 0.49 | 0.43 ± 0.49 | −0.10 (−0.23, 0.02) | 0.088 |
| Week 8 | 0.27 ± 0.38 | 0.34 ± 0.53 | −0.06 (−0.18, 0.05) | 0.297 |
| Daily total, number of 15-minute periods | | | | |
| Week 1 | 3.49 ± 2.67 | 3.82 ± 2.81 | −0.33 (−0.98, 0.31) | 0.312 |
| Week 2 | 3.14 ± 2.73 | 4.09 ± 2.70 | −0.91 (−1.55, −0.26) | **0.006** |
| Week 4 | 2.20 ± 2.10 | 3.13 ± 2.21 | −0.88 (−1.41, −0.35) | **0.001** |
| Week 8 | 2.28 ± 1.83 | 2.74 ± 1.94 | −0.45 (−0.92, 0.03) | 0.066 |

*Note:* Data are shown as mean ± standard deviation. Group differences were calculated using analysis of covariance, adjusted for age, sex, and average daily formula intake in the past week. Bold text denotes statistical significance (*p* < 0.05).

Abbreviations: A1PF, infant formula made from milk free of A1-type β-casein; CI, confidence interval; CON, infant formula made from conventional milk (containing both A1- and A2-type β-casein).

**Supporting Information: Table 5.** Infants’ Dermatitis Quality of Life index scores (intention-to-treat population)

|  | **A1PF group *n* = 140** | | **CON group *n* = 140** | | ***p*-value** |
| --- | --- | --- | --- | --- | --- |
|  | **Median (IQR)** | **Range** | **Median (IQR)** | **Range** |  |
| **Dermatitis severity** | | | | | |
| Baseline | 0 (0–1) | [0–2] | 0 (0–1) | [0–2] | 0.292 |
| Week 1 | 0 (0–1) | [0–2] | 0 (0–1) | [0–2] | 0.425 |
| Week 2 | 0 (0–1) | [0–2] | 0 (0–1) | [0–2] | 0.779 |
| Week 4 | 0 (0–0) | [0–2] | 0 (0–1) | [0–3] | 0.188 |
| Week 8 | 0 (0–0) | [0–2] | 0 (0–1) | [0, 4] | 0.350 |
| **Life quality index** | | | | | |
| 1. Itching and scratching | | | | | |
| Baseline | 0 (0–1) | [0–2] | 0 (0–1) | [0–1] | 0.358 |
| Week 1 | 0 (0–1) | [0–2] | 0 (0–1) | [0–2] | 0.285 |
| Week 2 | 0 (0–1) | [0–2] | 0 (0–1) | [0–3] | 0.397 |
| Week 4 | 0 (0–0) | [0–2] | 0 (0–1) | [0–3] | 0.552 |
| Week 8 | 0 (0–0) | [0–2] | 0 (0–1) | [0–3] | 0.165 |
| 2. Mood | | | | | |
| Baseline | 0 (0–1) | [0–2] | 0 (0–1) | [0–3] | 0.601 |
| Week 1 | 0 (0–1) | [0–3] | 0 (0–1) | [0–3] | 0.103 |
| Week 2 | 0 (0–0) | [0–2] | 0 (0–1) | [0–3] | 0.177 |
| Week 4 | 0 (0–0) | [0–1] | 0 (0–1) | [0–2] | 0.095 |
| Week 8 | 0 (0–0) | [0–2] | 0 (0–0) | [0–3] | 0.229 |
| 3. Time to get to sleep | | | | | |
| Baseline | 1 (0–1) | [0–3] | 1 (0–1) | [0–3] | 0.900 |
| Week 1 | 1 (0–1) | [0–3] | 1 (0–1) | [0–3] | 0.687 |
| Week 2 | 1 (0–1) | [0–3] | 1 (0–1) | [0–3] | 0.778 |
| Week 4 | 0 (0–1) | [0–2] | 0.5 (0–1) | [0–3] | 0.560 |
| Week 8 | 0 (0–1) | [0–2] | 0 (0–1) | [0–2] | 0.715 |
| 4. Sleep disturbances | | | | | |
| Baseline | 0 (0–0) | [0–3] | 0 (0–0) | [0–2] | 0.698 |
| Week 1 | 0 (0–0) | [0–2] | 0 (0–0) | [0–1] | 0.169 |
| Week 2 | 0 (0–0) | [0–2] | 0 (0–0) | [0–2] | 0.080 |
| Week 4 | 0 (0–0) | [0–2] | 0 (0–0) | [0–2] | 0.121 |
| Week 8 | 0 (0–0) | [0–2] | 0 (0–0) | [0–2] | 0.441 |
| 5. Disturbed playing or swimming | | | | | |
| Baseline | 0 (0–0) | [0–1] | 0 (0–0) | [0–1] | 0.777 |
| Week 1 | 0 (0–0) | [0–1] | 0 (0–0) | [0–1] | 0.154 |
| Week 2 | 0 (0–0) | [0–1] | 0 (0–0) | [0–1] | 0.538 |
| Week 4 | 0 (0–0) | [0–1] | 0 (0–0) | [0–2] | 0.250 |
| Week 8 | 0 (0–0) | [0–1] | 0 (0–0) | [0–3] | 0.135 |
| 6. Disturbed family activities | | | | | |
| Baseline | 0 (0–0) | [0–1] | 0 (0–0) | [0, 1] | 0.556 |
| Week 1 | 0 (0–0) | [0–1] | 0 (0–0) | [0–1] | 0.286 |
| Week 2 | 0 (0–0) | [0–1] | 0 (0–0) | [0–2] | 0.334 |
| Week 4 | 0 (0–0) | [0–1] | 0 (0–0) | [0, 1] | 0.200 |
| Week 8 | 0 (0–0) | [0–1] | 0 (0–0) | [0–3] | 0.072 |
| 7. Problems during mealtimes | | | | | |
| Baseline | 0 (0–0) | [0–1] | 0 (0–0) | [0–1] | 0.643 |
| Week 1 | 0 (0–0) | [0–1] | 0 (0–0) | [0–1] | 0.555 |
| Week 2 | 0 (0–0) | [0–1] | 0 (0–0) | [0–1] | 0.667 |
| Week 4 | 0 (0–0) | [0–1] | 0 (0–0) | [0–1] | 0.418 |
| Week 8 | 0 (0–0) | [0–1] | 0 (0–0) | [0–3] | 0.730 |
| 8. Problems from treatment | | | | | |
| Baseline | 0 (0–0) | [0–1] | 0 (0–0) | [0–1] | 0.652 |
| Week 1 | 0 (0–0) | [0–1] | 0 (0–0) | [0–1] | 0.497 |
| Week 2 | 0 (0–0) | [0–1] | 0 (0–0) | [0–1] | 0.410 |
| Week 4 | 0 (0–0) | [0–1] | 0 (0–0) | [0–1] | 0.264 |
| Week 8 | 0 (0–0) | [0–1] | 0 (0–0) | [0–3] | 0.754 |
| 9. Dressing problems | | | | | |
| Baseline | 0 (0–0) | [0–1] | 0 (0–0) | [0, 1] | 0.556 |
| Week 1 | 0 (0–0) | [0–1] | 0 (0–0) | [0, 1] | 0.318 |
| Week 2 | 0 (0–0) | [0–1] | 0 (0–0) | [0, 1] | 0.573 |
| Week 4 | 0 (0–0) | [0–1] | 0 (0–0) | [0, 1] | 0.200 |
| Week 8 | 0 (0–0) | [0–1] | 0 (0–0) | [0–2] | 0.149 |
| 10. Problems at bath time | | | | | |
| Baseline | 0 (0–0) | [0–1] | 0 (0–0) | [0–1] | 0.520 |
| Week 1 | 0 (0–0) | [0–1] | 0 (0–0) | [0–1] | 0.370 |
| Week 2 | 0 (0–0) | [0–1] | 0 (0–0) | [0–1] | 0.777 |
| Week 4 | 0 (0–0) | [0–1] | 0 (0–0) | [0–2] | 0.537 |
| Week 8 | 0 (0–0) | [0–1] | 0 (0–0) | [0–2] | 0.200 |
| **Total IDQoL score** | | | | | |
| Baseline | 1 (1–3) | [0–12] | 2 (1–3) | [0–9] | 0.338 |
| Week 1 | 1 (0–2) | [0–10] | 1 (0.5–3) | [0–11] | 0.084 |
| Week 2 | 1 (0–2) | [0–10] | 1 (0–3) | [0–9] | 0.084 |
| Week 4 | 1 (0–2) | [0–10] | 1 (0–2) | [0–12] | 0.318 |
| Week 8 | 1 (0–2) | [0–12] | 1 (0–2) | [0–25] | 0.243 |

Abbreviations: A1PF, infant formula made from milk free of A1-type β-casein; CON, infant formula made from conventional milk (containing both A1- and A2-type β-casein); IDQoL, Infants’ Dermatitis Quality of Life index; IQR, interquartile range.

**Supporting Information: Table 6.** Infants’ Dermatitis Quality of Life index scores among participants with family history of allergies (intention-to-treat population)

|  | **A1PF group *n* = 140** | | **CON group**  ***n* = 140** | | ***p*-value** |
| --- | --- | --- | --- | --- | --- |
|  | **Median (IQR)** | **Range** | **Median (IQR)** | **Range** |  |
| **Dermatitis severity** | | | | | |
| Baseline | 0 (0–1) | [0–2] | 0 (0–1) | [0–2] | 0.309 |
| Week 1 | 0 (0–1) | [0–2] | 0 (0–1) | [0–2] | 0.857 |
| Week 2 | 0 (0–1) | [0–2] | 0 (0–1) | [0–2] | 0.977 |
| Week 4 | 0 (0–0) | [0–2] | 0 (0–1) | [0–3] | 0.196 |
| Week 8 | 0 (0–0) | [0–2] | 0 (0–1) | [0–4] | 0.102 |
| **Life quality index** | | | | | |
| 1. Itching and scratching | | | | | |
| Baseline | 0 (0–1) | [0–2] | 0 (0–1) | [0–1] | 0.122 |
| Week 1 | 0 (0–1) | [0–1] | 0 (0–1) | [0–2] | 0.857 |
| Week 2 | 0 (0–1) | [0–2] | 0 (0–1) | [0–1] | 0.977 |
| Week 4 | 0 (0–0) | [0–2] | 0 (0–1) | [0–3] | 0.196 |
| Week 8 | 0 (0–0) | [0–2] | 0 (0–1) | [0–3] | 0.125 |
| 2. Mood | | | | | |
| Baseline | 0 (0–1) | [0–1] | 1 (0–1) | [0–3] | 0.295 |
| Week 1 | 0 (0–1) | [0–3] | 0 (0–1) | [0–3] | 0.238 |
| Week 2 | 0 (0–1) | [0–2] | 0 (0–1) | [0–2] | 0.184 |
| Week 4 | 0 (0–0) | [0–1] | 0 (0–1) | [0–2] | 0.270 |
| Week 8 | 0 (0–0) | [0–2] | 0 (0–0) | [0–3] | 0.182 |
| 3. Time to get to sleep | | | | | |
| Baseline | 1 (0–1) | [0–3] | 1 (0–1) | [0–3] | 0.525 |
| Week 1 | 1 (0–1) | [0–3] | 1 (0–1) | [0–3] | 0.870 |
| Week 2 | 1 (0–1) | [0–3] | 0 (0–1) | [0–3] | 0.710 |
| Week 4 | 0 (0–1) | [0–1] | 0 (0–1) | [0–3] | 0.330 |
| Week 8 | 0 (0–1) | [0–2] | 0 (0–1) | [0–2] | 0.897 |
| 4. Sleep disturbances | | | | | |
| Baseline | 0 (0–0) | [0–3] | 0 (0–0) | [0–2] | 0.807 |
| Week 1 | 0 (0–0) | [0–2] | 0 (0–0) | [0–1] | 0.522 |
| Week 2 | 0 (0–0) | [0–1] | 0 (0–0) | [0–1] | 0.409 |
| Week 4 | 0 (0–0) | [0–2] | 0 (0–0) | [0–2] | 0.103 |
| Week 8 | 0 (0–0) | [0–2] | 0 (0–0) | [0–2] | 0.320 |
| 5. Disturbed playing or swimming | | | | | |
| Baseline | 0 (0–0) | [0–1] | 0 (0–0) | [0–1] | 0.458 |
| Week 1 | 0 (0–0) | [0–1] | 0 (0–0) | [0–1] | 0.575 |
| Week 2 | 0 (0–0) | [0–1] | 0 (0–0) | [0–1] | 0.744 |
| Week 4 | 0 (0–0) | [0–1] | 0 (0–0) | [0–2] | 0.347 |
| Week 8 | 0 (0–0) | [0–1] | 0 (0–0) | [0–3] | 0.082 |
| 6. Disturbed family activities | | | | | |
| Baseline | 0 (0–0) | [0–1] | 0 (0–0) | [0–1] | 0.771 |
| Week 1 | 0 (0–0) | [0–1] | 0 (0–0) | [0–1] | 0.378 |
| Week 2 | 0 (0–0) | [0–1] | 0 (0–0) | [0–2] | 0.481 |
| Week 4 | 0 (0–0) | [0–1] | 0 (0–0) | [0–1] | 0.105 |
| Week 8 | 0 (0–0) | [0–1] | 0 (0–0) | [0–3] | 0.210 |
| 7. Problems during mealtimes | | | | | |
| Baseline | 0 (0–0) | [0–1] | 0 (0–0) | [0–1] | 0.674 |
| Week 1 | 0 (0–0) | [0–1] | 0 (0–0) | [0–1] | 0.536 |
| Week 2 | 0 (0–0) | [0–1] | 0 (0–0) | [0–1] | 0.352 |
| Week 4 | 0 (0–0) | [0–1] | 0 (0–0) | [0–1] | 0.382 |
| Week 8 | 0 (0–0) | [0–1] | 0 (0–0) | [0–3] | 0.496 |
| 8. Problems from treatment | | | | | |
| Baseline | 0 (0–0) | [0–1] | 0 (0–0) | [0–1] | 0.944 |
| Week 1 | 0 (0–0) | [0–1] | 0 (0–0) | [0–1] | 0.352 |
| Week 2 | 0 (0–0) | [0–1] | 0 (0–0) | [0–1] | 0.072 |
| Week 4 | 0 (0–0) | [0–1] | 0 (0–0) | [0–1] | 0.479 |
| Week 8 | 0 (0–0) | [0–1] | 0 (0–0) | [0–3] | 0.974 |
| 9. Dressing problems | | | | | |
| Baseline | 0 (0–0) | [0–1] | 0 (0–0) | [0–1] | 0.458 |
| Week 1 | 0 (0–0) | [0–1] | 0 (0–0) | [0–1] | 0.251 |
| Week 2 | 0 (0–0) | [0–1] | 0 (0–0) | [0–1] | 0.304 |
| Week 4 | 0 (0–0) | [0–1] | 0 (0–0) | [0–1] | 0.208 |
| Week 8 | 0 (0–0) | [0–1] | 0 (0–0) | [0–2] | 0.327 |
| 10. Problems at bath time | | | | | |
| Baseline | 0 (0–0) | [0–1] | 0 (0–0) | [0–1] | 0.116 |
| Week 1 | 0 (0–0) | [0–1] | 0 (0–0) | [0–1] | 0.304 |
| Week 2 | 0 (0–0) | [0–1] | 0 (0–0) | [0–1] | 0.557 |
| Week 4 | 0 (0–0) | [0–1] | 0 (0–0) | [0–2] | 0.900 |
| Week 8 | 0 (0–0) | [0–1] | 0 (0–0) | [0–2] | 0.376 |
| **Total IDQoL score** | | | | | |
| Baseline | 1 (1–3) | [0–12] | 2 (1–3) | [0–9] | 0.070 |
| Week 1 | 1 (0–2) | [0–10] | 1 (0–3) | [0–11] | 0.267 |
| Week 2 | 1 (0–2) | [0–10] | 1 (0–3) | [0–9] | 0.274 |
| Week 4 | 1 (0–2) | [0–10] | 1 (0–3) | [0–12] | 0.284 |
| Week 8 | 0 (0–2) | [0–12] | 1 (0–3) | [0, 25] | 0.136 |

Abbreviations: A1PF, infant formula made from milk free of A1-type β-casein; CON, infant formula made from conventional milk (containing both A1- and A2-type β-casein); IDQoL, Infants’ Dermatitis Quality of Life index; IQR, interquartile range.

**Supporting Information: Table 7.** Average daily amount of formula feeding and other food intake in the past week (intention-to-treat population)

|  | **A1PF group**  ***n* = 140** | **CON group**  ***n* = 140** | **Group difference (A1PF versus CON)** | |
| --- | --- | --- | --- | --- |
|  |  |  | **Difference of least-squares means (95% CI)** | ***p*-value** |
| Formula feedings, times/day | | | | |
| Week 1 | 5.05 ± 0.34 | 5.01 ± 0.39 | 0.05 (−0.04, 0.14) | 0.253 |
| Week 2 | 5.00 ± 0.33 | 4.98 ± 0.29 | 0.03 (−0.04, 0.10) | 0.413 |
| Week 4 | 4.85 ± 0.37 | 4.90 ± 0.34 | −0.04 (−0.13, 0.04) | 0.340 |
| Week 8 | 4.77 ± 0.29 | 4.83 ± 0.37 | −0.04 (−0.12, 0.03) | 0.274 |
| Breastfeeding, times/day | | | | |
| Week 1 | 1.71 ± 0.33 | 1.76 ± 0.37 | −0.05 (−0.13, 0.04) | 0.258 |
| Week 2 | 1.56 ± 0.35 | 1.59 ± 0.30 | −0.03 (−0.11, 0.05) | 0.451 |
| Week 4 | 1.48 ± 0.32 | 1.50 ± 0.32 | −0.02 (−0.10, 0.06) | 0.647 |
| Week 8 | 1.48 ± 0.30 | 1.45 ± 0.32 | 0.02 (−0.06, 0.10) | 0.602 |
| Total infant formula intake, mL/day | | | | |
| Week 1 | 499.47 ± 39.84 | 495.90 ± 40.21 | 3.79 (−5.72, 13.29) | 0.434 |
| Week 2 | 508.50 ± 34.35 | 506.31 ± 27.68 | 2.22 (−5.18, 9.61) | 0.556 |
| Week 4 | 555.88 ± 37.21 | 564.04 ± 35.28 | −7.71 (−16.53, 1.12) | 0.087 |
| Week 8 | 638.97 ± 40.63 | 645.74 ± 46.83 | −3.83 (−14.13, 6.48) | 0.465 |
| Feeding duration, min/day | | | | |
| Week 1 | 103.95 ± 4.81 | 104.00 ± 3.86 | 0.07 (−0.96, 1.09) | 0.899 |
| Week 2 | 100.89 ± 4.32 | 101.22 ± 4.25 | −0.22 (−1.22, 0.79) | 0.674 |
| Week 4 | 98.41 ± 4.88 | 99.07 ± 4.85 | −0.48 (−1.64, 0.68) | 0.419 |
| Week 8 | 97.03 ± 5.08 | 97.36 ± 5.49 | −0.04 (−1.31, 1.23) | 0.951 |

*Note:* Data are shown as mean ± standard deviation. Group differences were calculated using analysis of covariance, adjusted for age, sex, and average daily formula intake in the past week.

Abbreviations: A1PF, infant formula made from milk free of A1-type β-casein; CI, confidence interval; CON, infant formula made from conventional milk (containing both A1- and A2-type β-casein).

**Supporting Information: Table 8.** Adverse events (intention-to-treat population)

|  | **A1PF group**  **(*n* = 140)** | **CON group**  **(*n* = 140)** | **Overall**  **(*n* = 280)** |
| --- | --- | --- | --- |
| Total number of AEs | 35 | 38 | 73 |
| Number of participants with any AE^†^ | 31 (22.14) | 31 (22.14) | 62 (22.14) |
| Ear, eyes, nose, throat |  |  |  |
| Ear eczema | 1 | 0 | 1 |
| Gastrointestinal |  |  |  |
| Diarrhea | 1 | 2 | 3 |
| Constipation | 4 | 5 | 9 |
| Lack of appetite | 2 | 3 | 5 |
| Respiratory |  |  |  |
| Upper respiratory infection^†^ | 6 | 5 | 11 |
| Cold^†^ | 2 | 5 | 7 |
| Cough without other related symptoms | 7 | 4 | 11 |
| Skin |  |  |  |
| Diaper rash | 0 | 2 | 2 |
| Eczema | 4 | 6 | 10 |
| Other |  |  |  |
| Fever | 8 | 6 | 14 |

*Note:* Data are shown as n (%) or number of events.

^†^*p* = 1.000, Fisher’s exact test

Abbreviations: A1PF, infant formula made from milk free of A1-type β-casein; AE, adverse event; CON, infant formula made from conventional milk (containing both A1- and A2-type β-casein).

^†^Recorded based on the physician’s diagnosis.
